# Monoclonal Antibodies in Treating Food Allergy: A New Therapeutic Horizon

**DOI:** 10.3390/nu13072314

**Published:** 2021-07-05

**Authors:** Sara Manti, Giulia Pecora, Francesca Patanè, Alessandro Giallongo, Giuseppe Fabio Parisi, Maria Papale, Amelia Licari, Gian Luigi Marseglia, Salvatore Leonardi

**Affiliations:** 1 Pediatric Respiratory Unit, Department of Clinical and Experimental Medicine, San Marco Hospital, University of Catania, Via Santa Sofia 78, 95123 Catania, Italy; saramanti@hotmail.it (S.M.); giupec87@hotmail.it (G.P.); francescapatane27@gmail.com (F.P.); giuseppeparisi88@hotmail.it (G.F.P.); mariellapap@yahoo.it (M.P.); leonardi@unict.it (S.L.); 2 Pediatric Clinic, Department of Pediatrics, Fondazione IRCCS Policlinico San Matteo, University of Pavia, 27100 Pavia, Italy; amelialicari@gmail.com (A.L.); glmarseglia@smatteo.pv.it (G.L.M.)

**Keywords:** monoclonal antibodies, food allergy, biologics, children, adults

## Abstract

Food allergy (FA) is a pathological immune response, potentially deadly, induced by exposure to an innocuous and specific food allergen. To date, there is no specific treatment for FAs; thus, dietary avoidance and symptomatic medications represent the standard treatment for managing them. Recently, several therapeutic strategies for FAs, such as sublingual and epicutaneous immunotherapy and monoclonal antibodies, have shown long-term safety and benefits in clinical practice. This review summarizes the current evidence on changes in treating FA, focusing on monoclonal antibodies, which have recently provided encouraging data as therapeutic weapons modifying the disease course.

## 1. Introduction

Food Allergy (FA) is a pathological and potentially deadly immune response caused by exposure to an innocuous and specific food allergen [1]. Epidemiological global data suggest that FA prevalence ranges from 0.45% to 10% among children younger than five years old. It has been estimated that approximately 40% of patients with FA have experienced a life-threatening allergic reaction, and that 30% of children with FA show multiple FAs [2,3].

Based on the underlying immune mechanism, FA is broadly classified into immunoglobulin (Ig)E-mediated (characterized by immediate reactions), non-IgE mediated (characterized by delayed reactions), or mixed (characterized by both IgE-dependent and IgE-independent mechanisms). The main characteristics of IgE-mediated, non-IgE mediated, and mixed FAs are summarized in Table 1.

Affecting up to 10% of the pediatric population [4], IgE-mediated FA is the most common and costly FA subtype. Although the allergens triggering the FA vary with country and dietary habits, milk, egg, peanut, wheat, soy, and shellfish are currently the most common foods to induce IgE-mediated FA [5]. After exposure to the offending allergen, food allergen-derived epitopes bind to the IgE and, by binding with the FcεRI receptor expressed on the surface of mast cells and basophils, induce the IgE-mediated degranulation of the immune effector cells. The latter releases preformed histamine, leukotrienes (LTs), platelet-activating factor (PAF), and cytokines such as interleukin-4 (IL-4), IL-5, and IL-13, which are able to maintain the allergic immune response [1]. Clinically, early and rapid symptom onset can occur and may involve one or more systems among the cutaneous system (with flushing, urticaria, angioedema, pruritus), respiratory system (with bronchial hyperresponsiveness and/or wheezing), gastrointestinal system (with nausea, abdominal pain, diarrhea, and vomiting), and cardiovascular system (with hypotension). Whenever a severe allergic reaction involves multiple organ systems, the patient experiences anaphylaxis, which can potentially become a life-threatening event [1,2,3,4,5,6].

In addition to the classic IgE-mediated FA, two variants are worthy of consideration: oral allergy syndrome (OAS) and FA to the carbohydrate galactose-alpha-1,3-galactose (alpha-gal). OAS is characterized by the immediate onset of oral pruritus, mucosal angioedema, and/or abdominal pain in patients with allergic rhinitis who produce specific IgE for aero-allergens cross-reactive with fruit- or vegetable-protein epitopes. As plant-derived proteins are also sensitive to heat exposure, the same foods are typically tolerated after cooking. This aspect can help in diagnosing OAS [7,8].

The FA to alpha-gal occurs in patients producing specific IgE for the red meat carbohydrate alpha-gal after exposure to tick vectors Dermacentor variabilis (brown dog tick) and Amblyomma americanum (lone star tick). Although it is an IgE-mediated FA, unlike the classic IgE-mediated allergic reactions, the FA to the carbohydrate alpha-gal features a delayed reaction and lacks any relationship with other atopic diseases. Other mechanisms behind the type-2 immune response have been suggested to be involved in the pathogenesis of FA to alpha-gal [8].

Food protein-induced enterocolitis syndrome (FPIES), food protein-induced allergic proctocolitis (FPIAP), and food protein enteropathy (FPE) are the most widely known non-IgE-mediated FAs [9]. Generally, the median age at FPIES onset is 5.5 months. In accordance with the symptom onset, clinical features, duration and severity of symptoms, and offending foods, FPIES is classified into early- (primarily within three months of age) and late-onset (mostly four to seven months of age); typical or atypical type (in older patients, positive skin prick test results, and serum-specific IgE levels); acute and chronic symptoms; and milk or soy FPIES, solid food FPIES, and multiple food FPIES. Clinically, FPIES is primarily characterized by gastrointestinal symptoms, such as profuse vomiting, sometimes accompanied by diarrhea; however, a variable and atypical clinical presentation can also occur. FPIES can occur after the first or second ingestion of the offensive food, as a result of an inappropriate T-cell activation and proliferation, leading to the release of pro-inflammatory cytokines, such as tumor necrosis factor-a (TNF-a), interferon-y (IFN-y), IL-8, and IL-9, which, in turn, impair the permeability barrier, inducing local intestinal inflammation [10].

FPIAP is characterized by inflammatory injury in the distal colon in response to one or more offending food proteins, such as cow’s milk or soy. Studies provide evidence that failure in Th3 cells, low levels of transforming growth factor β (TGF-β), and high expression of TNF-a may be involved in the pathogenesis of this disease. Patients affected by FPIAP generally present with red blood and mucus mixed with the stool, with or without diarrhea; they are generally healthy in appearance and do not report weight loss. Diagnosis is clinical, and FPIAP diagnosis is confirmed when patients respond positively to eliminating a suspected triggering food allergen after excluding other causes of gastrointestinal symptoms, such as necrotizing enterocolitis, intussusception, infectious colitis, anal fissures, and very early onset inflammatory bowel disease [11].

FPE is mainly characterized by non-bloody diarrhea, malabsorption, and failure to thrive in the first nine months of life [9]. It is triggered in formula-fed infants, but also by soybean, wheat, and egg. Diagnostic tests are not available, and diagnosis is based on clinical history, physical examination, and an oral food challenge (OFC) [9].

Mixed FAs include Eosinophilic Gastrointestinal Disorders (EGIDs), such as eosinophilic esophagitis (EoE), allergic eosinophilic gastroenteritis (AEG), and eosinophilic colitis, characterized by gastrointestinal symptoms, eosinophilic infiltration of the gastrointestinal tract, and, sometimes, peripheral eosinophilia [12]. Generally detected in the first year of life, the clinical picture of EoE includes regurgitation, vomiting, rumination, lack of appetite, burning, and pain, causing crying after feeding and sometimes immediately after starting to feed. The suspicion of EoE increases when the response to a proton pump inhibitor (PPI) is lacking. The esophageal biopsy shows a diagnostic eosinophilic infiltration (>15 eosinophils per high-power field (eos/hpf) [13].

Multiple food allergens are implicated in the onset of AEG [14], generally affecting children and adults. According to the severity of the involvement of bowel wall layers, abdominal pain, irritability, vomiting, diarrhea, weight loss, easy satiety, anemia, and hypoalbuminemia range from a mild to a severe degree. The esophageal biopsy shows eosinophilic infiltration of gastric and/or duodenal mucosa (>30 eos/hpf). Moreover, in approximately 50% of patients with AEG, peripheral eosinophilia, positive food skin prick tests (SPTs), and specific IgE antibodies can be found [15].

Eosinophilic colitis is the less common of the EGIDs. It is generally seen in adolescents affected by inflammatory bowel disease and/or celiac disease and allergy to cow’s milk protein, soya, or peanuts. Currently, there is no consensus on the diagnosis of eosinophilic colitis; however, the detection of >50 eos/hpf in the ascending colon, >42 eos/hpf in the transverse and descending colon, and >32 eos/hpf in the rectosigmoid colon are considered suggestive of eosinophilic colitis [16]. 

The main clinical characteristics of FAs are summarized in Table 1.

**Figure 1:**
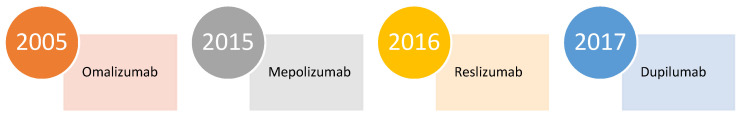
Developmental timing of monoclonal antibodies used for treating allergic disorders.

Regardless of their pathogenesis and clinical pictures, and due to the lack of definitive treatment, FAs represent a significant burden on affected children and their families, due to dietary restrictions, diet adherence, fear of accidental reactions, and the self-management of anaphylactic reactions. To date, no specific treatment for FAs is available, so their therapeutic management is limited to dietary avoidance. However, allergen-specific therapy (immunotherapy) is showing encouraging results. In parallel, several therapeutic strategies are also emerging that restore immune tolerance against the offending food epitopes (Figure 1). In this regard, treatment with monoclonal antibodies has recently provided encouraging results as a therapeutic weapon modifying the disease course.

## 2. Monoclonal Antibodies in FA

### 2.1. Omalizumab

The critical mediator involved in FA is IgE, making it a promising therapeutic target. As a prototype of an anti-IgE, omalizumab is a humanized IgG1 monoclonal antibody that acts through various mechanisms. Firstly, it binds to free IgEs, blocking them from binding with specific high-affinity receptors (FcεRI) expressed on dendritic and mast cells. Furthermore, it decreases receptor expression on these cells, thus interfering upstream with the inflammatory cascade. It also leads to a reduction of IgE synthesis by B-cells. At present, anti-IgE therapy is one of the mainstay treatments for severe asthma, severe chronic urticaria, and severe chronic rhinosinusitis with nasal polyps (CRSwNP) [17,18]. Omalizumab is administered as a subcutaneous injection; the dosage, time interval, and frequency are based on a nomogram derived from baseline total serum IgE levels and body weight (kilograms). The following section is focused on the available literature on anti-IgE therapy for the management of FA, where anti-IgE is still off label [19].

Eighty-four individuals, aged between 12 and 60 years old, affected by peanut allergy, were enrolled in a double-blind, placebo-controlled, randomized clinical trial (RCT) to test the anti-IgE monoclonal antibody TNX-901. Patients were randomized into four groups, and three different doses of TNX-901 (150 mg, 300 mg, 450 mg) or a placebo were administered for four monthly doses. Two to four weeks after the end of treatment, the subjects underwent an OFC, which showed a significant increase of threshold dose for peanut, compared to screening at enrollment, only in those receiving 450 mg of TNX-901. Nevertheless, 25% of the patients did not develop tolerance to peanuts, evidencing variable responses among them [20].

Regarding children, an RCT including patients aged 6–13 years old raised safety concerns due to the reactions to pre-omalizumab challenges and, therefore, was stopped early [21].

An open-label study enrolling 14 individuals aged between 18 and 50 years with a history of peanut allergy evaluated the effectiveness of a six-month treatment with anti-IgE. The median threshold tolerated dose for peanut significantly increased from an 80 mg baseline to 6500 mg after treatment. However, the study had some limitations due to the small sample size (*n* = 14 adults) and the need for antihistamines and epinephrine in 10 out of 14 patients at the third food challenge, after six months of omalizumab [22].

To maximize the development of tolerance and reduce safety concerns relating to immunotherapy, a synergic effect of combined therapy with anti-IgE and FA-AIT was hypothesized. Accordingly, 13 children, with a median age of 10 years, suffering from peanut allergy, were enrolled in a double-blind, placebo-controlled food challenge trial. Children underwent a course of omalizumab combined with rapid oral food allergy desensitization. Omalizumab was administered during the 12 weeks before and during oral food desensitization, until a maintenance oral dose of peanut (8000 mg) was reached. Following the peanut challenge, 92% of patients tolerated an 8000 mg dose of peanut flour, and 39% reported moderate to severe adverse reactions [23].

Another food challenge trial was conducted on 11 children with cow’s milk allergy. After nine weeks of omalizumab pretreatment, 9 out of 11 subjects completed an OFC and received omalizumab until week 16. Few reactions were reported (1.6% of cow’s milk doses administered), and most were mild [24].

Combined treatment with oral immunotherapy (OIT) and omalizumab has also been investigated in the setting of multiple FAs, in a phase I clinical trial enrolling 25 children (median age: 7 years) who were treated with OIT, up to five allergens, and omalizumab. The omalizumab was started eight weeks before the OIT. The safety outcome was satisfactory: reactions followed only 5.3% of administered doses, and 94% of these were mild. Only one child showed a severe reaction, and was treated with epinephrine [25].

The previously mentioned results were consistent with a subsequent phase II RCT of 48 patients aged 4–15 years with multiple FAs. Sixteen weeks of omalizumab treatment was significantly associated with a higher percentage of tolerance to up to 2 gr of at least two foods at 36 weeks, compared to a placebo (83% vs. 33%, *p* = 0.004), in a double-blind placebo-controlled food challenge (DBPCFC). Furthermore, omalizumab, compared with a placebo, significantly increased the tolerated dose, reduced the time taken to achieve a maintenance dose, and reduced the median rate of adverse reactions (27% vs. 68%), with no severe adverse events reported [26].

Contrary to previous studies, an RCT involving 57 patients aged 7–32 years with severe cow’s milk allergy did not significantly improve the success rate of OIT in those treated with omalizumab vs. a placebo over 28 months. However, omalizumab allowed patients to achieve a maintenance dose with fewer OIT doses and improved the safety of the OIT. Indeed, the incidence of adverse reactions was significantly lower (2.1% vs. 16.1% of doses, *p* = 0.0005) and those that did occur were less severe in the omalizumab group (2 vs. 18 doses requiring epinephrine) [27].

These findings were consistent with a study of 14 children aged between 4 months and 11 years affected by egg and cow’s milk allergies. The OIT was tolerated by all patients only if pretreatment and concomitant treatment with omalizumab took place. Nevertheless, a question arises about when omalizumab should be stopped. Indeed, six patients developed grade 3–4 anaphylactic symptoms after suspending omalizumab, suggesting the need for longer maintenance therapy with an anti-IgE [28].

As regards the underlying mechanism of omalizumab-induced desensitization, Bedoret et al. suggested that milk-specific CD4-T cells might be involved in the development of anergy. It has been suggested that a combination of omalizumab and oral desensitization with higher doses of milk is associated with an early reduction in the proliferation of T-CD4 milk-specific cells, through the development of anergy [29]. The underlying mechanism might be mediated by a reduction in antigen presentation induced by omalizumab [30]. Further, long-term desensitization was found to be associated with an increase in IFN/IL-4 ratio and IgG4, showing a shift in immune response, whose mechanism is still unclear. IgG4 could act by inhibiting IgE [29].

In conclusion, these data support the role of omalizumab as a viable therapeutic option in patients with FA through raising the threshold tolerance dose, thus reducing the risk of severe adverse reactions in the case of accidental ingestion [23,24,25,26,27,28,29,31]. Long-term follow-up studies are probably needed to strengthen these data. Indeed, only one study showed that, one year after the suspension of omalizumab, some patients relapsed and their specific IgE significantly reduced, although IgE levels could not be associated with the response to therapy or relapse [28].

As yet, omalizumab has not been approved as a treatment for FA, and the optimal dosage has not been determined. Basophil allergen threshold sensitivity has been suggested as a monitoring marker of response to omalizumab in patients with severe peanut allergy, and it might be helpful in individualizing therapy [32]. Currently, several ongoing trials investigate the role of omalizumab as a monotherapy or in combination with OIT. The clinical development program for omalizumab as a monotherapy or an adjunctive treatment in FA is summarized in Table 2 and Table 3.

### 2.2. Ligelizumab

Ligelizumab, also called QGE031, is a new humanized monoclonal anti-IgE antibody. It is administered as a subcutaneous injection at a dosage of 24, 72, or 240 mg every two weeks. It was initially tested in a phase II RCT, parallel design, dose-ranging, multi-center trial enrolling adult patients (age range, 18–50 years) affected by peanut allergy [56]. However, no results have been posted, as the recruitment was stopped.

### 2.3. Etokimab

Etokimab, also known as ANB020 (AnaptysBio), is a monoclonal antibody directed against IL-33, a pro-inflammatory cytokine that promotes B-class switching to IgE. The terminal half-life of etokimab is approximately 372 hours, with comparable values across all doses (10–750 mg) and regardless of route (i.v. or s.c.) of administration. In a six-week placebo-controlled phase II clinical trial enrolling 15 adults (age range, 19–54 years) with FAs, the authors showed that etokimab was safe and well tolerated. A single administration of etokimab as a monotherapy was able to induce immune tolerance to peanut, as well as reduce atopy-related adverse events in the enrolled patients [57].

### 2.4. Dupilumab

Dupilumab is a fully human IgG4 monoclonal antibody directed against the interleukin (IL)-4 receptor alpha (IL-4Ra) subunit, blocking IL-4- and IL-13-mediated pathways. By binding to IL-4Ra, a subunit also shared with the IL-13 receptor (IL-13R), dupilumab blocks the Th2-mediated inflammatory cascade [58,59]. Currently, dupilumab is approved in Europe for treating adolescents aged over 12 years, affected by severe asthma with an eosinophilic phenotype, or with oral corticosteroid-dependent asthma. The drug is available in prefilled syringes and is administered subcutaneously, once at a dose of 400 mg, then at 200 mg every two weeks; or once at 600 mg, then 300 mg every two weeks. The latter scheme is approved for patients who have oral corticosteroid-dependent asthma or comorbid moderate-to-severe atopic dermatitis (AD), for which dupilumab is indicated. Dupilumab is also indicated as an add-on maintenance treatment in patients older than 18 years with inadequately controlled CRSwNP [59,60]. The positive results of dupilumab studies in allergic diseases such as asthma, AD, and CRSwNP suggest that this monoclonal antibody can positively affect the course of other atopic diseases, including FA. Rial et al. [60] reported the first evidence of the efficacy of dupilumab in treating FAs in a 30-year-old woman with a positive history of severe AD and allergic rhinitis without asthma. Several ongoing clinical trials are evaluating dupilumab as either a monotherapy or an adjunct to oral immunotherapy for peanut allergy. Specifically, the NCT04462055 trial [54] is a three-year observational clinical trial to evaluate the effect of dupilumab on change in clinical eliciting dose (i.e., the lowest dose causing an allergic reaction) in subjects with peanut, hazelnut, walnut, cow’s milk, hen’s egg, and/or soybean allergy. It was conducted in a cohort of 21 patients (≥12 years) with moderate-to-severe AD. This study is still ongoing, and no preliminary results have been published. The NCT04394351 trial [55], a prospective phase II, single-center trial, is currently ongoing, and no preliminary results have been published. In this RCT, the authors aim to demonstrate the efficacy of dupilumab, compared to a placebo, in treating 110 pediatric patients, aged 6 to 21 years, with active EoE and multiple allergies. The efficacy of the dupilumab treatment will be assessed via endoscopic visual measurements of disease activity using the Eosinophilic Esophagitis-Endoscopic Reference Score (EoE-EREFS) and histologic abnormalities as measured by the EoE Histology Scoring System (EoE-HSS). Clinical trials on dupilumab’s use in treating FAs are summarized in Table 3 and Table 4.

## 3. Conclusions

The high prevalence of FA, its impact on quality of life, and the risk of life-threatening reactions have highlighted the need for new treatment strategies other than avoidance of the involved food allergen alone [62]. Although OIT has shown promising results, the use of monoclonal antibodies in treating FA has been suggested based on the pathogenic mechanism. Various trials have highlighted the role of monoclonal antibodies, both as monotherapies and in combination with OIT, in improving the threshold of tolerated dose of allergens. Therefore, monoclonal antibodies may emerge as a more effective, tailored, and potentially disease-modifying therapy for FA. Nevertheless, the application of monoclonal antibodies in food allergy treatment is rather novel and not many well-controlled, large-sample-size studies are available to date; therefore, updated reviews of the literature need to be carried out on a regular basis as more data are published.

## Figures and Tables

**Table 1:** Main clinical findings of Food Allergies in pediatric population.

Ig-E Mediated Food Allergies					
Disorder	World Prevalence	Common Allergens	Description	Diagnosis	Treatment
Urticaria/angioedema Contact urticaria	Up to 14.5% for males and 16.2% for females 13.3–24.5%	Milk, egg, peanut, nuts, fish, shellfish Fresh fruit, fish, milk, egg	Immediate reaction to foods with erythema and wheals Urticaria resulting from direct contact with skin	SPT*, serum IgE * levels, and OFC *	Elimination diet and emergency medication Research: OIT *, SLIT *, EPIT *, and biologic drugs Contact avoidance and emergency medication
Oral allergy syndrome	5–8%	Fresh fruits and vegetables	Itching and mild edema of oral cavity	SPT or PBP *, serum IgE levels, and OFC	Elimination diet and emergency medication Research: OIT, SLIT, EPIT, and biologic drugs
Anaphylaxis	0.3%	Milk, egg, peanuts, nuts, fish, shellfish	Rapid reaction with involvement of skin, respiratory tract, and cardiocirculatory apparatus	SPT, serum IgE levels, and OFC	Elimination diet and emergency medication Research: OIT, SLIT, EPIT, and biologic drugs
Exercise-induced anaphylaxis	5–15%	Wheat, shellfish, celery	Food induces anaphylaxis only if ingestion is temporally followed by physical exercise	Anamnesis	Elimination diet, time interval between food consumption and exercise, and emergency medication Research: OIT, SLIT, EPIT, and biologic drugs
**Non IgE-Mediated Food Allergies**					
**Disorder**	**Prevalence**	**Common Allergens**	**Description**	**Diagnosis**	**Treatment**
FPIES Food protein-induced proctocolitis	Few data Few data	Milk, egg, soy, oat, rice Milk protein through breast feeding or egg, soy, wheat	Immediate reaction to foods with vomiting, diarrhea, pallor, sweating, hypotension Mucus in stools	Clinical history and OFC Elimination diet and OFC	Elimination diet and drugs Elimination diet
Food protein enteropathy	Few data	Milk, egg, soy, and wheat	Malabsorption syndrome	Elimination diet or OFC with jejunal biopsy	Elimination diet
**Mixed Food Allergies**					
**Disorder**	**Prevalence**	**Common Allergens**	**Description**	**Diagnosis**	**Treatment**
Atopic dermatitis Eosinophilic esophagitis	27–37% of patients with AD * Up to 50/100,000 patients	Mostly milk and egg Egg, milk, beef, chicken, soy, and wheat	Immediate reaction to foods with erythema and wheals Reflux symptoms including vomiting, dysphagia, cough, and food impaction	SPT, serum IgE levels, and OFC Eosinophil infiltrates on esophageal biopsies	Elimination diet Research: OIT, SLIT, EPIT, and biologic drugs Elimination diet or topical steroids
Eosinophilic gastroenteritis	Rare	Multiple allergens or may not have food allergy etiology	Nonspecific gastrointestinal disorders associated with eosinophilic infiltrate of gastrointestinal tract region and layer	Eosinophil infiltrates on gastrointestinal biopsies, eosinophils in ascites	Elimination diet or topical steroids

* SPT: skin prick test; IgE: immunoglobulin E; PBP: prick by prick; OFC: oral food challenge; OIT: oral immunotherapy; SLIT: Sublingual-swallow immunotherapy; EPIT: Epicutaneous Immunotherapy; AD: atopic dermatitis.

**Table 2:** Randomized Clinical Trials for omalizumab as monotherapy or as adjunctive treatment in food allergy.

	Number Clinical Trial	Status	Phase	Estimated Enrollment (No. Patients)	Patients’ Age (Years)	Primary Outcome	Drugs	Drug Dosage	Results
1	NCT02879006 [33]	Ongoing	2	34	≥6 and ≤40 *	Sustained unresponsiveness	Chinese herbal medication, placebo, omalizumab, multi-OIT*	Not applicable	Not yet reported
2	NCT02643862 [34]	Concluded	2	48	≥4 and ≤55	Desensitization assessed by proportion of FA * individuals who tolerate a DBPCFC * up to 2000 mg protein for each of 2 allergens at week 36	Omalizumab, placebo	Not applicable	Not yet reported
3	NCT03181009 [35]	Ongoing	2	60	≥2 and ≤25	Change in allergen-specific serum IgG4 * and IgE *	Omalizumab, food flour allergens	Omalizumab: subjects ≥4 years receive 150 mg. Subjects ≤4 years receive 75 mg Food flour allergens: 300 to 1200 mg	Not yet reported
4	NCT02626611 [36]	Concluded	2	70	≥4 and ≤55	No. individuals tolerating an OFC to 2000 mg for at least 2 allergens at week 36	Omalizumab, food flour buildup	Omalizumab: not applicable Food flour buildup: up to 2000mg	Not yet re-ported
5	NCT01510626 [37]	Concluded	1	35	≥4 and ≤55	No. adverse events in the treatment group	Omalizumab food protein	Not applicable	Not yet reported
6	NCT00949078 [38]	Concluded	2	51	≥18 and ≤50	1. No. patients who experienced a decrease in Pn-BHR * AUC * of > 80% compared with baseline values before week 8 2. Percentage change in peanut-specific IgE from baseline to after Pn-BHR response 3. Percentage change in peanut-specific IgE after pn-BHR response 4. Total IgE after pn-BHR response 5. Dose of peanut protein inducing allergic symptoms at OFC*1 6. Dose of peanut protein inducing allergic symptoms at OFC2 7. Dose of peanut protein inducing allergic symptoms at OFC3 8. Omalizumab received before OFC2 9. No. doses of omalizumab received before OFC2	Omalizumab, food allergen	Not applicable	Not yet reported
7	NCT01781637 [39]	Not yet started	1, 2	36	≥7 and ≤25	Tolerance of 2000 mg 6 weeks after last dose of omalizumab/placebo	Omalizumab, placebo	Not applicable	Not yet reported
8	NCT03881696 [40]	Not yet started	3	225	≥2 and ≤55	No. participants by stage 1 treatment group, omalizumab versus placebo, who successfully consumed ≥600 mg of peanut protein without dose-limiting symptoms during the DBPCFC conducted at the end of treatment stage 1	Omalizumab, placebo, multi-allergen OIT	Omalizumab: 75 to 150 mg	Not yet reported
9	NCT02402231 [41]	Not yet started	2	23	≥12 and ≤22	Peanut challenge	Omalizumab, immunotherapy	Not applicable	Not yet reported
10	NCT01157117 [42]	Concluded	2	77	≥7 and ≤35	Percentage of subjects in omalizumab group vs. placebo group developing clinical tolerance to milk	Omalizumab, milk powder	Omalizumab: not applicable, milk powder: up to 3.84 g	Omalizumab vs. milk powder: *p* = 0.42
11	NCT00968110 [43]	Concluded	1	10	≥4 and ≤18	To assess the safety of omalizumab in young children, and the safety of oral desensitization in patients pretreated with omalizumab	Omalizumab	Not applicable	Not yet reported
12	NCT00086606 [44]	Concluded	2	150	≥6 and ≤75	Not applicable	Omalizumab	Not applicable	Not yet reported
13	NCT00932282 [45]	Concluded	1, 2	13	≥12	Percentage of subjects who pass the 20gm peanut flour (~50% peanut protein) OFC 2–4 weeks after discontinuing peanut OIT therapy	Peanut OIT, omalizumab	Peanut OIT: 0.2 mg of peanut flour to 8000 mg omalizumab: not applicable	Not yet reported
14	NCT00382148 [46]	Concluded	2	10	≥6 and ≤75	Serious adverse events	Omalizumab	Not applicable	Not yet reported
15	NCT01290913 [47]	Concluded	1, 2	13	≥7 and ≤25	No. participants that tolerated rapid oral peanut desensitization to a dose of 500 mg peanut flour	Omalizumab	Not applicable	Not yet reported
16	NCT04045301 [48]	Ongoing	2	90	≥2 and ≤75	To evaluate the efficacy of Omalizumab at reducing time-to-maintenance during a symptom-driven multi-food OIT protocol	Omalizumab, placebo, multi-food OIT	Omalizumab 16 mg/kg, omalizumab 8 mg/kg	Not yet reported
17	NCT04037176 [49]	Ongoing	4	100	≥6 and ≤18	Change in challenge threshold after 3 months of treatment in patients treated with omalizumab vs. placebo	Omalizumab, placebo	Not applicable	Not yet reported
18	NCT01040598 [50]	Concluded	1	19	12 to 76	To assess markers that will predict responders to Omalizumab	Omalizumab	Not applicable	Not applicable
19	NCT00123630 [51]	Concluded	2	30	12 to 60	Change in eosinophil numbers per high power field proximally and distally between baseline and post-treatment and between both groups	Omalizumab, placebo	Omalizumab or placebo: 150 to 375 mg SC * every 2 or 4 weeks.	Not applicable
20	NCT00084097 [52]	Concluded	2	30	≥12 and ≤70	To evaluate safety of omalizumab and its efficacy in reducing peripheral blood absolute eosinophil count pre- and post-administration of omalizumab	Omalizumab	maximum dose of 375 mg every 2 weeks	Not yet reported
21	NCT03964051 [53]	Ongoing	4	10	≥18 and ≤70	Change in food challenge threshold	Omalizumab	300 mg every 2 weeks for 12 weeks	Not yet reported

* N.: number; pts: patients; OIT: oral immunotherapy; FA: food allergy; DBPCFC: double-blind placebo-controlled food challenge; IgG: Immunoglobulin-G; IgE: Immunoglobulin-E; Pn-BHR: peanut allergen induced basophil histamine release; AUC: Area under curve; OFC: oral food challenge; SC: subcutaneously.

**Table 3:** Clinical development program for dupilumab as monotherapy or as adjunctive treatment in food allergy.

	Number Clinical Trial	Status	Phase	Estimated Enrollment (No. Patients)	Patients’ Age (Years)	Primary Outcome	Drugs	Drug Dosage	Results
1	NCT04462055 [54]	Ongoing	Not stated	21	≥12	To determine the effect of dupilumab on change in clinical eliciting dose (i.e., lowest dose causing an allergic reaction) in subjects with peanut, hazelnut, walnut, cow’s milk, hen’s egg and/or soybean allergy	Dupilumab	Not applicable	Not applicable
2	NCT04394351 [55]	Ongoing	3	90	≥1 and ≤11	Proportion of patients achieving peak esophageal intraepithelial eosinophil count ≤ 6 eos/hpf (400×)	Dupilumab, placebo	Not applicable	Not applicable

**Table 4:** Clinical development program for Omalizumab and Dupilumab as treatment in food allergy.

	Number Clinical Trial	Status	Phase	Estimated Enrollment (No. Patients)	Patients’ Age (Years)	Primary Outcome	Drugs	Drug Dosage	Results
1	NCT03679676 [61]	Not started	2	200	≥6 and ≤21	The success rates of passing a peanut food challenge	Omalizumab, placebo, dupilumab	Not applicable	Not applicable

## Data Availability

Not applicable.

## References

[1] Boyce J.A., Assa’ad A., Burks A.W., Jones S.M., Sampson H.A., Wood R.A., Plaut M., Cooper S.F., Fenton M.J., Arshad S.H. (2010). Guidelines for the diagnosis and management of food allergy in the United States: Summary of the NIAID-sponsored Expert Panel Report. J. Allergy Clin. Immunol..

[2] Prescott S.L., Pawankar R., Allen K.J., Campbell D.E., Sinn J.K., Fiocchi A., Ebisawa M., Sampson H.A., Beyer K., Lee B.-W. (2013). A global survey of changing patterns of food allergy burden in children. World Allergy Organ. J..

[3] Gupta R.S., Springston E.E., Warrier M.R., Smith B., Kumar R., Pongracic J., Holl J.L. (2011). The prevalence, severity, and distribution of childhood food allergy in the United States. Pediatrics.

[4] Osborne N.J., Koplin J.J., Martin P.E., Gurrin L.C., Lowe A.J., Matheson M.C., Ponsonby A.L., Wake M., Tang M.L., Dharmage S.C. (2011). Prevalence of challenge-proven IgE-mediated food allergy using population-based sampling and predetermined challenge criteria in infants. J. Allergy Clin. Immunol..

[5] Chinthrajah R.S., Tupa D., Prince B.T., Block W.M., Rosa J.S., Singh A.M., Nadeau K. (2015). Diagnosis of Food Allergy. Pediatr. Clin. N. Am..

[6] Cuppari C., Manti S., Salpietro A., Valenti S., Capizzi A., Arrigo T., Salpietro C., Leonardi S. (2016). HMGB1 levels in children with atopic eczema/dermatitis syndrome (AEDS). Pediatr. Allergy Immunol..

[7] Ivković-Jureković I. (2015). Oral allergy syndrome in children. Int. Dent. J..

[8] Wilson J.M., Schuyler A.J., Schroeder N., Platts-Mills T.A. (2017). Galactose-α-1,3-Galactose: Atypical Food Allergen or Model IgE Hypersensitivity?. Curr. Allergy Asthma Rep..

[9] Nowak-Węgrzyn A., Katz Y., Mehr S.S., Koletzko S. (2015). Non-IgE-mediated gastrointestinal food allergy. J. Allergy Clin. Immunol..

[10] Manti S., Leonardi S., Salpietro A., Del Campo G., Salpietro C., Cuppari C. (2017). A systematic review of food protein-induced enterocolitis syndrome from the last 40 years. Ann. Allergy Asthma Immunol..

[11] Mennini M., Fiocchi A.G., Cafarotti A., Montesano M., Mauro A., Villa M.P., Di Nardo G. (2020). Food protein-induced allergic proctocolitis in infants: Literature review and proposal of a management protocol. World Allergy Organ. J..

[12] Calvani M., Anania C., Cuomo B., D’Auria E., Decimo F., Indirli G., Marseglia G., Mastrorilli V., Sartorio M., Santoro A. (2021). Non-IgE- or Mixed IgE/Non-IgE-Mediated Gastrointestinal Food Allergies in the First Years of Life: Old and New Tools for Diagnosis. Nutrients.

[13] Hirano I., Furuta G.T. (2020). Approaches and Challenges to Management of Pediatric and Adult Patients with Eosinophilic Esophagitis. Gastroenterology.

[14] Abou Rached A., El Hajj W. (2016). Eosinophilic gastroenteritis: Approach to diagnosis and management. World J. GastroIntest. Pharmacol. Ther..

[15] Pajno G.B., Castagnoli R., Muraro A., Alvaro-Lozano M., Akdis C.A., Akdis M., Arasi S. (2019). Allergen immunotherapy for IgE-mediated food allergy: There is a measure in everything to a proper proportion of therapy. Pediatr. Allergy Immunol..

[16] DiTommaso L.A., Rosenberg C.E., Eby M.D., Tasco A., Collins M.H., Lyles J.L., Putnam P.E., Mukkada V., Rothenberg M.E. (2019). Prevalence of eosinophilic colitis and the diagnoses associated with colonic eosinophilia. J. Allergy Clin. Immunol..

[17] Giallongo A., Parisi G.F., Licari A., Pulvirenti G., Cuppari C., Salpietro C., Marseglia G.L., Leonardi S. (2019). Novel therapeutic targets for allergic airway disease in children. Drugs Context.

[18] Licari A., Manti S., Castagnoli R., Parisi G.F., Salpietro C., Leonardi S., Marseglia G.L. (2019). Targeted Therapy for Severe Asthma in Children and Adolescents: Current and Future Perspectives. Paediatr. Drugs.

[19] Pelaia C., Calabrese C., Terracciano R., de Blasio F., Vatrella A., Pelaia G. (2018). Omalizumab, the first available antibody for biological treatment of severe asthma: More than a decade of real-life effectiveness. Ther. Adv. Respir. Dis..

[20] Leung D.Y., Sampson H.A., Yunginger J.W., Burks A.W., Schneider L.C., Wortel C.H., Davis F.M., Hyun J.D., Shanahan W.R. (2003). Effect of anti-IgE therapy in patients with peanut allergy. N. Engl. J. Med..

[21] Sampson H.A., Leung D.Y., Burks A.W., Lack G., Bahna S.L., Jones S.M., Wong D.A. (2011). A phase II, randomized, double-blind, parallel-group, placebo-controlled oral food challenge trial of Xolair (omalizumab) in peanut allergy. J. Allergy Clin. Immunol..

[22] Savage J.H., Courneya J.P., Sterba P.M., Macglashan D.W., Saini S.S., Wood R.A. (2012). Kinetics of mast cell, basophil, and oral food challenge responses in omalizumab-treated adults with peanut allergy. J. Allergy Clin. Immunol..

[23] Schneider L.C., Rachid R., LeBovidge J., Blood E., Mittal M., Umetsu D.T. (2013). A pilot study of omalizumab to facilitate rapid oral desensitization in high-risk peanut-allergic patients. J. Allergy Clin. Immunol..

[24] Nadeau K.C., Schneider L.C., Hoyte L., Borras I., Umetsu D.T. (2011). Rapid oral desensitization in combination with omalizumab therapy in patients with cow’s milk allergy. J. Allergy Clin. Immunol..

[25] Bégin P., Dominguez T., Wilson S.P., Bacal L., Mehrotra A., Kausch B., Trela A., Tavassoli M., Hoyte E., O’Riordan G. (2014). Phase 1 results of safety and tolerability in a rush oral immunotherapy protocol to multiple foods using omalizumab. Allergy Asthma Clin. Immunol..

[26] Andorf S., Purington N., Block W.M., Long A.J., Tupa D., Brittain E., Spergel A.R., Desai M., Galli S.J., Nadeau K.C. (2018). Anti-IgE treatment with oral immunotherapy in multifood allergic participants: A double-blind, randomised, controlled trial. Lancet Gastroenterol. Hepatol..

[27] Wood R.A., Kim J.S., Lindblad R., Nadeau K., Henning A.K., Dawson P., Plaut M., Sampson H.A. (2016). A randomized, double-blind, placebo-controlled study of omalizumab combined with oral immunotherapy for the treatment of cow’s milk allergy. J. Allergy Clin. Immunol..

[28] Martorell-Calatayud C., Michavila-Gomez A., Martorell-Aragone’s A., Molini-Menchón N., Cerdá-Mir J.C., Félix-Toledo R., De Las Marinas-Álvarez M.D. (2016). Anti-IgE assisted desensitization to egg and cow’s milk in patients refractory to conventional oral immunotherapy. Pediatr. Allergy Immunol..

[29] Bedoret D., Singh A.K., Shaw V., Hoyte E.G., Hamilton R., DeKruy R.H., Schneider L.C., Nadeau K.C., Umetsu D.T. (2012). Changes in antigen-specific T-cell number and function during oral desensitization in cow’s milk allergy enabled with omalizumab. Mucosal. Immunol..

[30] Holgate S.T., Djukanović R., Casale T., Bousquet J. (2005). Anti-immunoglobulin E treatment with omalizumab in allergic diseases: An update on anti-inflammatory activity and clinical efficacy. Clin. Exp. Allergy.

[31] Licari A., Manti S., Marseglia A., De Filippo M., De Sando E., Foiadelli T., Marseglia G.L. (2020). Biologics in Children with Allergic Diseases. Curr. Pediatr Rev..

[32] Brandström J., Vetander M., Lilja G., Johansson S.G.O., Sundqvist A.-C., Kalm F., Nilsson C., Nopp A. (2017). Individually dosed omalizumab: An effective treatment for severe peanut allergy. Clin. Exp. Allergy.

[33] E-B-FAHF-2, Multi OIT and Xolair (Omalizumab) for Food Allergy. https://clinicaltrials.gov/ct2/show/NCT02879006.

[34] Study Using Xolair in Rush Multi Oral Immunotherapy in Multi Food Allergic Patients. https://clinicaltrials.gov/ct2/show/NCT02643862.

[35] Multi OIT to Test Immune Markers after Minimum Maintenance Dose. https://clinicaltrials.gov/ct2/show/NCT03181009.

[36] Multi Immunotherapy to Test Tolerance and Xolair. https://clinicaltrials.gov/ct2/show/NCT02626611.

[37] Omalizumab with Oral Food Immunotherapy with Food Allergies Open Label Safety Study in a Single Center. https://clinicaltrials.gov/ct2/show/NCT01510626.

[38] Omalizumab in the Treatment of Peanut Allergy. https://clinicaltrials.gov/ct2/show/NCT00949078.

[39] Peanut Reactivity Reduced by Oral Tolerance in an Anti-IgE Clinical Trial. https://clinicaltrials.gov/ct2/show/NCT01781637.

[40] Omalizumab as Monotherapy and as Adjunct Therapy to Multi-Allergen OIT in Food Allergic Participants. https://clinicaltrials.gov/ct2/show/NCT03881696.

[41] Treatment of Severe Peanut Allergy with Xolair (Omalizumab) and Oral Immunotherapy. https://clinicaltrials.gov/ct2/show/NCT02402231.

[42] OIT and Xolair^®^ (Omalizumab) in Cow’s Milk Allergy. https://clinicaltrials.gov/ct2/show/NCT01157117.

[43] Xolair Treatment for Milk Allergic Children. https://clinicaltrials.gov/ct2/show/NCT00968110.

[44] A Safety and Efficacy Study of Xolair in Peanut Allergy. https://www.clinicaltrials.gov/ct2/show/NCT00086606.

[45] Peanut Oral Immunotherapy and Anti-Immunoglobulin E (IgE) for Peanut Allergy. https://clinicaltrials.gov/ct2/show/NCT00932282.

[46] A Study of Xolair in Peanut-Allergic Subjects Previously Enrolled in Study Q2788g. https://clinicaltrials.gov/ct2/show/NCT00382148.

[47] Xolair Enhances Oral Desensitization in Peanut Allergic Patients. https://clinicaltrials.gov/ct2/show/NCT01290913.

[48] Omalizumab to Accelerate a Symptom-Driven Multi-Food OIT. https://clinicaltrials.gov/ct2/show/NCT04045301.

[49] Behandling af Boern Med Foedevareallergi Med Omalizumab (Xolair). https://clinicaltrials.gov/ct2/show/NCT04037176.

[50] Identifying Responders to Xolair (Omalizumab) Using Eosinophilic Esophagitis as a Disease Model. https://clinicaltrials.gov/ct2/show/NCT01040598.

[51] A Pilot Study of the Treatment of Eosinophilic Esophagitis with Omalizumab. https://www.clinicaltrials.gov/ct2/show/NCT00123630.

[52] Omalizumab to Treat Eosinophilic Gastroenteritis. https://clinicaltrials.gov/ct2/show/NCT00084097.

[53] Protection from Food Induced Anaphylaxis by Reducing the Serum Level of Specific IgE (Protana). https://clinicaltrials.gov/ct2/show/NCT03964051.

[54] Effectiveness of Dupilumab in Food Allergic Patients with Moderate to Severe Atopic Dermatitis. https://clinicaltrials.gov/ct2/show/NCT04462055.

[55] Study to Investigate the Efficacy and Safety of Dupilumab in Pediatric Patients with Active Eosinophilic Esophagitis (EoE). https://clinicaltrials.gov/ct2/show/NCT04394351.

[56] Efficacy and Safety of 4 Doses of QGE031 in Patients 18–50 Years of Age with Peanut Allergy. https://clinicaltrials.gov/ct2/show/NCT01451450.

[57] Chinthrajah S., Cao S., Liu C., Lyu S.-C., Sindher S.B., Long A., Sampath V., Petroni D., Londei M., Nadeau K.C. (2019). Phase 2a randomized, placebo-controlled study of anti-IL-33 in peanut allergy. JCI Insight.

[58] (2020). Dupilumab FDA Prescribing Information. https://www.accessdata.fda.gov/drugsatfda_docs/label/2019/761055s014lbl.pdf.

[59] Dupilumab E.M. (2020). Summary of Product Characteristics. https://www.ema.europa.eu/en/documents/product-information/dupixent-epar-product-information_en.pdf.

[60] Rial M.J., Barroso B., Sastre J. (2019). Dupilumab for treatment of food allergy. J. Allergy Clin. Immunol. Pract..

[61] Clinical Study Using Biologics to Improve Multi OIT Outcomes. https://clinicaltrials.gov/ct2/show/NCT03679676.

[62] Calvani M., Anania C., Caffarelli C., Martelli A., Miraglia Del Giudice M., Cravidi C., Duse M., Manti S., Tosca M.A., Cardinale F. (2020). Food allergy: An updated review on pathogenesis, diagnosis, prevention and management. Acta Bio-Med. Atenei Parm..

